# Cholesteryl Ester Transfer Protein (*CETP*) Polymorphisms Affect mRNA Splicing, HDL Levels, and Sex-Dependent Cardiovascular Risk

**DOI:** 10.1371/journal.pone.0031930

**Published:** 2012-03-05

**Authors:** Audrey C. Papp, Julia K. Pinsonneault, Danxin Wang, Leslie C. Newman, Yan Gong, Julie A. Johnson, Carl J. Pepine, Meena Kumari, Aroon D. Hingorani, Philippa J. Talmud, Sonia Shah, Steve E. Humphries, Wolfgang Sadee

**Affiliations:** 1 Program in Pharmacogenomics, Department of Pharmacology, College of Medicine, The Ohio State University, Columbus, Ohio, United States of America; 2 Department of Pharmacy Practice, College of Pharmacy, University of Florida, Gainesville, Florida, United States of America; 3 College of Medicine, Division of Cardiovascular Medicine, University of Florida, Gainesville Florida, United States of America; 4 Genetic Epidemiology Group, Department of Epidemiology and Public Health, University College London, London, United Kingdom; 5 Centre for Cardiovascular Genetics, BHF Laboratories, Institute Cardiovascular Science, University College London, London, United Kingdom; 6 UCL Genetics Institute, University College London, London, United Kingdom; FuWai Hospital - Chinese Academy of Medical Sciences, China

## Abstract

Polymorphisms in and around the Cholesteryl Ester Transfer Protein (*CETP*) gene have been associated with HDL levels, risk for coronary artery disease (CAD), and response to therapy. The mechanism of action of these polymorphisms has yet to be defined. We used mRNA allelic expression and splice isoform measurements in human liver tissues to identify the genetic variants affecting CETP levels. Allelic *CETP* mRNA expression ratios in 56 human livers were strongly associated with several variants 2.5–7 kb upstream of the transcription start site (*e.g.*, rs247616 p = 6.4×10^−5^, allele frequency 33%). In addition, a common alternatively spliced *CETP* isoform lacking exon 9 (Δ9), has been shown to prevent CETP secretion in a dominant-negative manner. The Δ 9 expression ranged from 10 to 48% of total *CETP* mRNA in 94 livers. Increased formation of this isoform was exclusively associated with an exon 9 polymorphism rs5883-*C>T* (p = 6.8×10^−10^) and intron 8 polymorphism rs9930761-*T>C* (5.6×10^−8^) (in high linkage disequilibrium with allele frequencies 6–7%). rs9930761 changes a key splicing branch point nucleotide in intron 8, while rs5883 alters an exonic splicing enhancer sequence in exon 9.

The effect of these polymorphisms was evaluated in two clinical studies. In the Whitehall II study of 4745 subjects, both rs247616 and rs5883*T*/rs9930761*C* were independently associated with increased HDL-C levels in males with similar effect size (rs247616 p = 9.6×10^−28^ and rs5883 p = 8.6×10^−10^, adjusted for rs247616). In an independent multiethnic US cohort of hypertensive subjects with CAD (INVEST-GENE), rs5883*T*/rs9930761*C* alone were significantly associated with increased incidence of MI, stroke, and all-cause mortality in males (rs5883: OR 2.36 (CI 1.29–4.30), p = 0.005, n = 866). These variants did not reach significance in females in either study. Similar to earlier results linking low CETP activity with poor outcomes in males, our results suggest genetic, sex-dependent *CETP* splicing effects on cardiovascular risk by a mechanism independent of circulating HDL-C levels.

## Introduction

CETP shuttles cholesterol esters from high-density lipoprotein particles (HDL) to low density lipoproteins (LDL). High CETP activity lowers the HDL/total cholesterol ratio, potentially increasing risk for coronary artery disease (CAD). Therefore, inhibition of CETP offers a new approach to CAD therapy [1], [2]. However, the CETP inhibitor torcetrapib was found to increase cardiovascular events, even though HDL increased and LDL decreased substantially [3]. As LDL supports reverse cholesterol transport to the liver, patients with rare genetic defects in CETP present with numerous lipid abnormalities [4]. Recent results further question the validity of the CETP-HDL-CAD relationship under all conditions, showing that low CETP levels can associate with increased CAD risk [5], possibly because of functions other than cholesterol transport.

*CETP* is highly polymorphic. Clinical studies have demonstrated a robust association of the *Taq1B* allele in intron 1 (rs708272*C>T*; *TaqIB_B_*, minor allele frequency (MAF) 0.44) with low CETP activity, decreased total cholesterol, and increased HDL cholesterol (HDL-C) [6]–[12]. In addition, *TaqIB_B_* was correlated with poor response to pravastatin in male but not female CAD patients [13]–[15]. While this finding has not been replicated in other studies [16]–[18], *CETP* polymorphisms appear to affect cardiovascular risk and therapy in a sex-dependent manner, reflecting different lipid metabolism in males and females [13]–[15]. However, *TaqIB* appears to serve merely as a surrogate marker for promoter/enhancer polymorphisms [19]–[25], and the responsible regulatory polymorphisms remain uncertain. Non-synonymous polymorphisms, such as rs5882*A>G* (I405V), have also been suggested to affect CETP function, but definitive data are lacking.

Alternative splicing also affects CETP activity. An in-frame deletion of exon 9 (Δ9) generates a shorter Δ9 protein, which dimerizes with the full-length form preventing its efflux from the liver [19], [26], possibly acting in a dominant negative manner. While production of the Δ9 splice variant is influenced by diet [27], [28], genetic factors have yet to be determined. To search for regulatory variants affecting CETP mRNA expression and test for the presence of genetic effects on splicing, we measured allelic mRNA expression and splicing in human livers, identifying candidate promoter/enhancer SNPs located 2.5–7 kb upstream, and discovering two SNPs in near complete linkage disequilibrium (LD) tightly associated with Δ9 CETP splicing. We then asked whether these polymorphisms affect HDL-C and risk for myocardial infarction.

## Results

### Total CETP mRNA levels in human livers and CETP genotype

PCR cycle thresholds (CTs; mean 27.9±1.1 SD) varied considerably for *CETP* mRNA between tissues. To scan the *CETP* locus for polymorphisms associated with mRNA expression, we genotyped multiple SNPs spanning ∼37 kb (Figure S1, Table S1). None of these SNPs yielded a robust association with overall mRNA levels, consistent with earlier results [23].

### Allelic CETP mRNA ratios in liver

**Figure 1:**
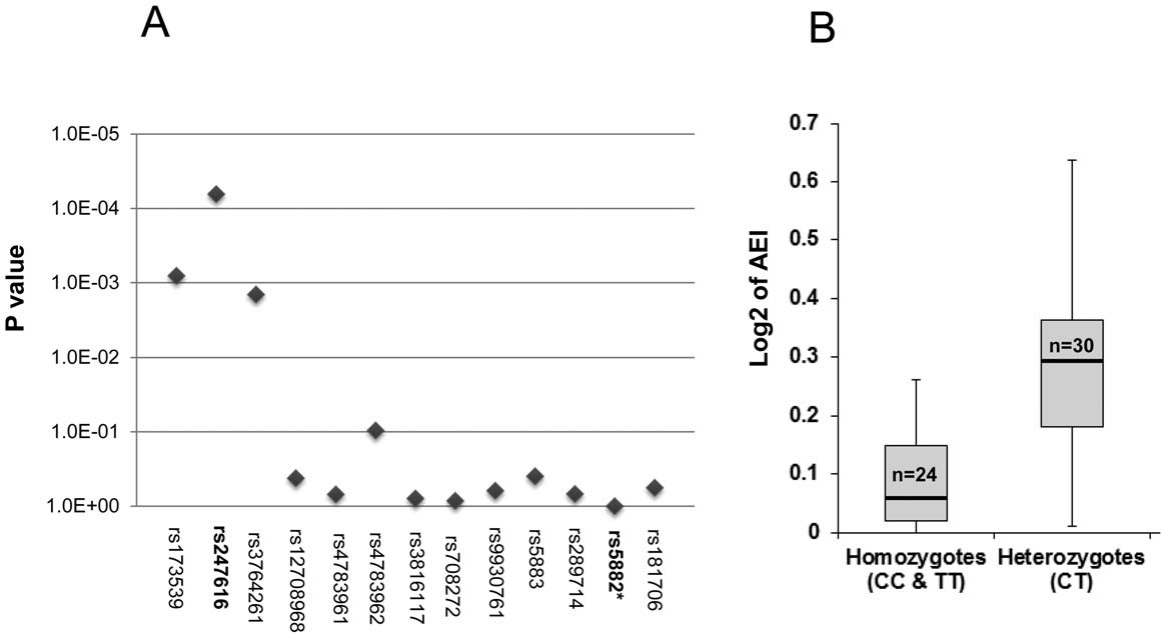
Association of CETP SNPs with Allelic Expression Ratios. Panel A. Association of each SNP with RNA absolute allelic ratios, as measured using rs5882 as an indicator. * Since rs5882 is used as the indicator in the assay, the p value is not applicable. The allelic mRNA ratios were normalized to the overall mean allelic gDNA ratios (there was no indication of a gene dosage effect requiring normalization to the gDNA for each individual). The data are mean ± S.D. (n = 3–6). Panel B. Log2 AEI absolute values in rs247616 genotypes. There would not be a detectable difference in allelic expression of homozygous samples if the SNP is functional.

Using the SNaPshot ™ primer extension assay, allelic mRNA ratios were measurable in 56 livers with rs5882 as the marker SNP. Significant allelic expression imbalance (AEI) was detectable in 29 of 56 livers tested (AEI ratios log_2_>0.4, or >30% below or above the mean gDNA ratio). The allelic mRNA ratios were distributed above and below the mean DNA ratio, indicating the presence of one or more *cis*-acting regulatory polymorphisms present in low LD with the marker SNP rs5882. Scanning the CETP locus with 13 SNPs genotyped in livers (Table S2), we determined SNP associations with presence of absence of AEI, or AEI ratios as continuous variable (absolute log ratios only). An LD structure map showing r^2^ and D′ values between all 13 SNPs is presented in Figure S5. Shown in Figure 1A, three SNPs located 2.6–7 kb upstream scored significantly (rs173539, rs247616, and rs3764261), with rs247616 having the strongest association (p = 6.4×10^−5^), indicating that transcription is under genetic control by these variants or others in high LD across the large 5′-haplotype block. Previously proposed promoter SNPs did not score significantly (Figure 1A). The allelic mRNA ratios differed strongly between genotypes of rs247616 (Figure 1B)

### Association of CETP exon 9 rs5883I*C>T* and intron 8 SNP rs9930761*T>C* with the Δ9 CETP mRNA splice variant in liver

**Figure 2:**
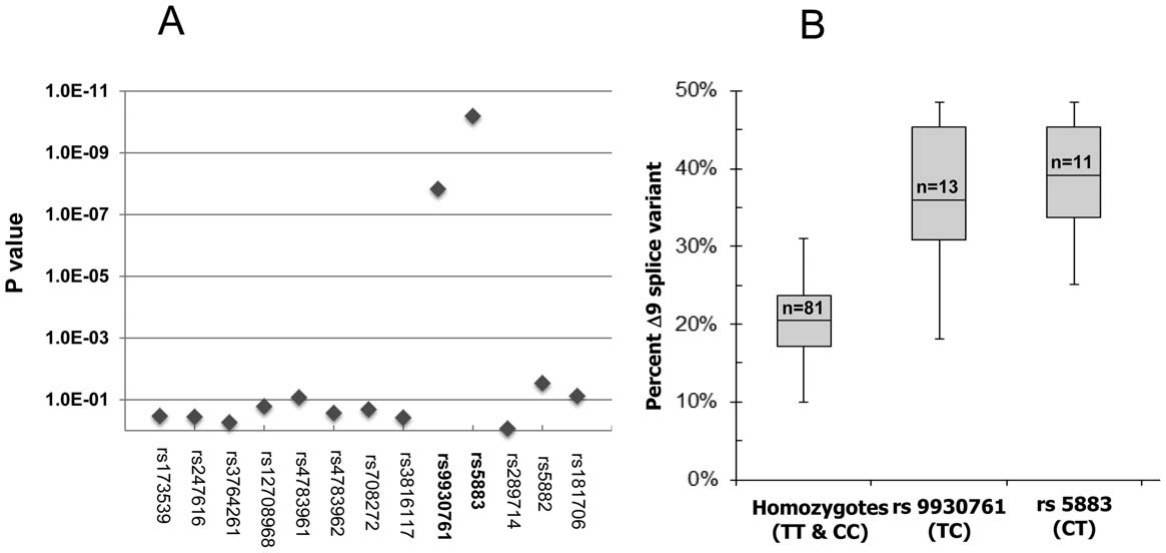
Association of CETP SNPs with the Δsplice variant. Panel A: Association p values assessing relationship between *CETP* SNPs and Δ9 splice variant formation in human livers (n = 94). Only rs9930761 and rs5883 in exon 8–10 region can account for increased formation of the Δ9 splice variant. rs289714 (intron 9), rs5882 (*I405V*), and rs1801706 (G84A) show varying degrees of LD with rs9930761, accounting for the observed association p values. Details for the SNPs in this study are provided in Table S3. Panel B: Percent Δ9 splice variant of total CETP mRNA as a function of rs5883*T>C* and rs9930761*C>T*. Homozygous minor allele carriers for rs5883/rs9930761 were not observed. All livers were heterozygous for both rs9930761 and rs5883, except for two livers heterozygous only for rs9930761, indicating that rs5883 is necessary for enhanced splicing. Using ANOVA with Dunnett's post-test, p values for both homozygous vs. rs9930761 and vs. rs5883 are P<0.01.

Measured with fluorescently labeled PCR primers, the Δ9 splice variant accounted for 10% to 48% of total CETP mRNA in 94 livers analyzed. An initial *CETP* SNP scan first revealed an association of I405V (rs5882) and *G84A* in the 3′UTR (rs1801706) with increased Δ9 splice variant, suggesting the presence of a splicing polymorphism. Sequencing a 3128 bp *CETP* genomic DNA region containing exon 8 through exon 10 in 6 liver tissues with low and high Δ9 splice variant expression yielded only two SNPs, in intron 8 (rs99300761*T>C*) and exon 9 (rs5883), present in all three tissues with high, and absent in those with low Δ9 expression. In 94 livers, rs5883 and rs9930761 (in complete LD (D′ = 1); MAF 5.9% and 6.9%%, respectively) were the only SNPs strongly associated with the Δ9 splice variant (p = 3.5E^−20^ and p = 1.7E^−17^, respectively) (Figure 2A). Levels of the Δ9 splice variant were markedly higher in rs5883*T* carriers (mean 39%, range 25–48% of total CETP mRNA) and in rs9930761*C* carriers (mean 36%, range 18–48%; compared to non-carriers (mean 20%, range 10–31%) (Figure 2B). There were no samples homozygous for the minor allele. Two subjects were heterozygous for rs9930761 but not rs5883. Both livers had a relatively low content of the Δ9 splice variant (18%), accounting for the lower p value of rs9930761 than of rs5883.

**Figure 3:**
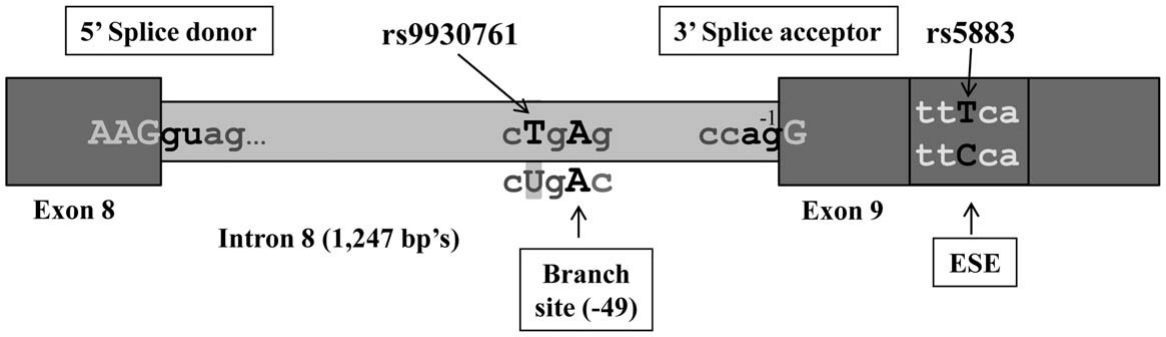
Schematics of the genomic CETP region spanning exons 8–10. The exonic enhancer site (ESE) in exon 9 is disrupted by rs5883. Splice site sequences and the predicted splice branch point with rs9930761 are also depicted.

Both rs9930761 and the synonymous SNP rs5883 have potential impact on the splicing process. The exonic rs5883 minor allele disrupts an exonic splicing enhancer site for SC35 (GTCTT**C**CA>GTCTT**T**CA) (ESE finder site (http://rulai.cshl.edu/cgi-bin/tools/ESE3/esefinder.cgi?process=home) (Figure 3). In addition, the rs5883 SNP alters predicted mRNA folding throughout exon 9, as calculated with Mfold (Figure S2). The minor *C* allele of rs9930761 disrupts a predicted splicing branch point (Figure 3).

### CETP haplotypes

While pair-wise LD analysis confirms the presence of two main 5′ and 3′ haplotypes blocks, using Helix Tree genetic analysis software package (Golden Helix, Inc., Bozeman, MT) [29] a 6-SNP haplotype analysis (rs173539, rs708272 (*Taq1B*), rs9930761 (or rs5883), rs5882 (*I405V*), and rs1801706 (*G84A*)) reveals the presence of only a few long-range haplotypes (Table S3). The minor *C*/*T* alleles of rs9930761/rs5883 are nearly exclusively embedded in a haplotype consisting of the wild-type alleles of *Taq1B* (intron 1) and rs173539 (upstream enhancer region), and the minor alleles of *I405V* (*G*) and *G84A* (*A*) accounting for weak associations of *I405V* and *G84A* with splicing (Figure 2A). Since the wild-type alleles of *Taq1B* and rs173539 (in high LD with rs247616) are associated with higher CETP levels and reduced HDL-C, the effects of rs5883/rs9930761 on HDL-C must be considered conditional on the upstream promoter SNPs.

### Association of rs5883/rs9930761 with HDL-C levels in Whitehall II

Of 95 *CETP* SNPs genotyped in 4,745 subjects, many SNPs were strongly associated with HDL-C, largely owing to high LD among them (e.g., rs247616 p = 7.18E^−29^), while, rs5883 and rs9930761 had lower significance (p = 6.09×10E^−6^, and p = 0.0012, respectively) (Table S4 A). The better score for rs5883 (Minor Allele Frequency 5.5%) versus rs9930761 (MAF 6.7%) in this cohort supports a critical role for rs5883 while a contribution from rs9930761 cannot be excluded. The lower overall significance for rs5883 and rs9930761 is partially accounted for by low allele frequency compared to enhancer region SNP rs247616 (33.6%). We then estimated associations of rs5883/rs9930761 with HDL-C by adjusting for rs247616, grouped by sex (females have higher HDL-C levels than males) (Table 1). The p values for both rs5883 and rs9930761 in males, when made contingent upon rs247616, decreased to p = 8.6E-10 and 3.8E-07, respectively (Table 1). Each minor allele of either rs5883/rs9930761 or rs247616 was independently associated with a substantial increase in HDL-C (∼0.1 mmol/L/minor allele) (Table S4 B), showing significant interactions between them (p = 0.00033; Table S4 C).

**Table 1:** Association analysis of HDL-C levels with rs9930761T>C and rs5883C>T, with and without adjusting for upstream SNP rs247616C>T, in the Whitehall II Study.

Male subjects					
SNP	position	Subject number	β	P	conditional on
rs247616	−6152 *C>T*	3332	0.072 (0.0065)	9.6E-28	-
rs9930761	Intron 8 *T>C*	3493	0.033 (0.012)	0.0078	-
rs9930761	Intron 8 *T>C*	3329	0.065 (0.013)	3.8E-07	rs247616
rs5883	Exon 9 *C>T*	3329	0.052(0.013)	8.6E-05	
rs5883	Exon 9 *C>T*	3329	0.084(0.014)	8.6E-10	rs247616

Female subjects					
rs247616	−6152 *C>T*	1181	0.067 (0.011)	9.2E-10	-
rs9930761	Intron 8 *T>C*	1247	0.024 (0.019)	0.20	-
rs9930761	Intron 8 *T>C*	1179	0.045 (0.019)	0.016	rs247616
rs5883	Exon 9 *C>T*	1181	0.047(0.021)	0.024	
rs5883	Exon 9 *C>T*	1181	0.069(0.021)	0.001	rs247616

Minor allele frequency (MAF) was 6.7% for rs9930761, 5.5% for rs5883, and 33.6% for rs247616. Linkage disequilibrium (LD) for rs247616–rs9930761 is R^2^ = 0.035 and D′ = 0.962 (haplotype frequencies are (versus expected under linkage equilibrium) TC 0.001 (0.023), CC 0.067 (0.044), TT 0.337 (0.315), and CT 0.595 (0.618). Note that the minor alleles rarely occur together (TC 0.001), indicating a strong negative LD between rs9930761/rs5883 and rs247616, requiring HDL-C versus rs9930761/rs5883 analysis conditional on rs247616. For all SNPs see Supporting Information Table S4.

### Effect of *CETP* rs5883/rs9930761 on risk of MI and other primary events in INVEST-GENES

This nested case–control study specifically tested the main hypothesis whether rs5883/rs9930761 affects risk of primary outcome events (cases: MI, stroke or all-cause mortality) in INVEST-GENES patients, in comparison to other regulatory variants that also increase HDL-C. With stratification by genotype, sex and race, significant associations were observed only in the Caucasian group (866 subjects, Table S5 A; other groups were too small). White male subjects, but not females, carrying the minor *rs5883T* and *rs9930761C* alleles (MAF 6.0% and 7.3%, respectively; D′ = 1,r^2^ = 0.88), had significantly increased risk of progression to first event (males p = 0.0018–0.0019, respectively, females p = 0.73–0.90) (Table 2, Table S5 A). The odds ratios for rs5883*T* and rs9930761*C* male carriers were 2.36 and 2.24, respectively (95% CI 1.29–4.30 and 1.28–3.91; p = 0.0051 and 0.008). Risk for white males without statin therapy was also substantial (OR 2.0; p = 0.034), but risk in the smaller statin-treated male group did not reach significance (rs9930761 carriers (OR 2.8; p = 0.089). Therefore, rs5883/rs9930761 appeared to be a general risk factor for male subjects, but larger cohorts are needed to assess the interaction with statin therapy.

**Table 2:** Associations between *CETP* rs9930761 and rs5883 minor variant carriers *versus* homozygous wild-type carriers, sex, race, and primary event (myocardial infarction, stroke or death) in INVEST.

group	rs9930761 Genotype	#	Odds ratio	95% CI	p value	SNP*statin interaction p value
White female	TT	347	(ref)			
	TC/CC	55	1.03	0.50–2.14	0.93	0.36
**White male**	TT	**395**	(ref)			
	TC/CC	**69**	**2.16**	**1.22–3.82**	**0.008**	0.9

group	rs5883 Genotype	#	Odds ratio	95% CI	p value	SNP*statin interaction p value
White female	CC	360	(ref)			
	CT/TT	43	1.02	0.45–2.35	0.96	0.23
**White male**	CC	**405**	(ref)			
	CT/TT	**59**	**2.36**	**1.29–4.30**	**0.0051**	0.64

Odds ratios represent occurrence of primary event for rs9930761/rs5883 carriers versus homozygous wild-type carriers (for all SNPs see Supporting Information Table S5 A).

The associations of additional *CETP* SNPs with outcomes are shown in Table S5 A, separated by males and females. Sex-dependent unadjusted p values of p<0.05 were observed for several SNPs, *e.g.*, enhancer region rs12708967 (males p = 0.012 and females p = 0.77). The same SNPs also showed highly significant associations HDL levels (rs12708967 p = 1.8E^−19^) (Table S5 B). However, some enhancer/promoter region SNPs scored only nominally significant in males and others in females (e.g., rs6499861), with p values that do not survive multiple hypotheses adjustments (necessary here because we have yet to identify the exact SNPs associated with transcription). Moreover, enhancer region rs247616 (strongly associated with AEI and HDL-C) failed to show significant association in INVEST-GENES (p = 0.592 in males and p = 0.067 in females). These results suggest that the promoter/enhancer SNPs did not show a detectable effect on outcomes in INVEST-GENES, in contrast to strong effects on HDL-C.

## Discussion

This study identifies two *CETP* SNPs strongly associated with splicing to a Δ9 CETP protein thought to act in a dominant-negative fashion. Both rs5883 and rs9930761 show significant associations with HDL and clinical outcomes in cardiovascular risk patients. Previously described *CETP* polymorphisms in a 5′ haplotype block affecting transcription also score highly with respect to HDL levels but failed to carry significant associations with clinical outcomes. Allelic CETP mRNA ratio analysis in human livers identified a region 2.5–7 kb 5′ upstream of the transcription start site, with at least three abundant SNPs, including rs247616, that are strong candidates as regulatory factors.

### Identification of promoter/enhancer SNPs affecting CETP mRNA expression

Using allelic CETP mRNA ratios measured in human livers, we have identified at least three upstream promoter/enhancer SNPs (rs173539, rs247616, and rs3764261) strongly associated with expression. Several of the SNPs tested here and previously proposed to have regulatory impact on transcription did not score significantly. This approach based on a highly reproducible proximate phenotype (allelic mRNA ratios) and SNP-scanning a gene locus has proven powerful for detecting regulatory variants [30]. Further extensive molecular studies are needed to determine which regulatory variant(s) modulate CETP expression, with multiple candidates contained in the 5′ haplotype block.

### rs5883/rs9930761 disrupts *CETP* mRNA splicing to yield the Δ9 splice variant

Formation of the Δ9 splice variant in human livers was associated with two SNPs in high LD (D′ = 1) with each other, intron 8 (rs9930761*T>C*; 5–7% allele frequency in Caucasians and ∼11% in subjects of African descent) and exon 9 (rs5883, with slightly lower minor allele frequency). rs5883 appears to be necessary for enhanced deletion of exon 9, judged by the relatively low Δ9 splice variant content in two livers heterozygous only for rs9930761 but not rs5883. However, all livers with high Δ9 splice variant content were heterozygous for both rs9930761 and rs5883, suggesting that both may be required to achieve effective skipping of exon 9. It is remarkable that the LD between the two SNPs is nearly complete even in African populations, residing predominantly in a single haplotype stretching over at least 20 kb, suggesting this represents an evolutionarily conserved haplotype.

The rs5883*T* allele disrupts an ESE enhancer consensus site and is predicted to alter RNA folding of the entire exon 9 (Figure S2). The rs9930761*C* allele, located 40 bp's upstream of exon 9, modulates a splicing branch point consensus sequence *C* ***T>C*** *RAY* required in mammalian splicing (Figure 3) [31]. With the intron 8 wild-type sequence *C* ***T*** *GAG* already predicted to be a weak branch point, low level of exon 9 skipping does occur in livers. Moreover, transfection of a minigene construct resulted in predominant exon 9 skipping (80–90%; data not shown), supporting the view that the splice branch point is already compromised in the wild-type sequence. As none of the livers were homozygous for the minor splicing allele, the maximum measured level of 48% Δ9 formation in heterozygotes represents a high degree of exon 9 skipping of the variant rs5883/rs9930761 alleles. No other *CETP* SNPs account for the observed genetic effect on splicing.

The biological effect of exon 9 deletion could be amplified by dominant-negative interactions through heterodimer formation of the Δ9 splice variant with full-length CETP, preventing cellular exit of mature CETP protein [19], [26]–[28]. Splicing in tissues other than the liver remains to be studied.

### Association of promoter/enhancer SNPs and rs5883/rs9930761 with HDL-C levels

A previous analysis of the Whitehall II study [25] showed that SNPs in CETP were predominantly associated with HDL-C and apoAI but less so or not at all with LDL-C, apoB, or TG. Strong HDL-C associations were observed with a series of promoter/enhancer SNPs present at high frequency (>30%) (*e.g.*, for rs247616 p = 6.14E^−29^), consistent with previous results [19]–[25], whereas the association was relatively weaker for rs9930761 and rs5883 (Table S4 A), seemingly indicating less clinical relevance. However, haplotype estimates revealed that rs5883*T*/rs9930761*C* predominantly share a haplotype consisting of the main wild-type alleles (associated with high HDL- levels) of all high scoring SNPs in the promoter/enhancer region (Table S3). Adjusting for enhancer SNP rs247616, the HDL-C association strengthened for both rs9930761 and rs5883 (p = 8.6E^−10^ in males) (Table 1). rs5883 consistently scored with greater significance than rs9930761, the latter with ∼1% greater allele frequency, supporting the notion that rs5883 is necessary for exon 9 skipping, while rs9930761 is insufficient but may also be required. A strong interaction was observed for effects on HDL-C between rs247616 and the splicing SNPs (interaction model p = 0.00033), consistent with their location on different haplotypes and mechanistically distinct effects. Considering the combined effects of rs247616 and rs5883 reveals that each minor allele appears to incrementally increase the HDL levels (Table S4 B). In particular, carriers heterozygous for both SNPs (n = 142) have substantially higher HDL (1.67 +/− 0.48 mmole/L compared to carriers of a minor allele in only one SNP (1.43 and 1.45 mmole/L) and carriers of only the main alleles (1.35 +/− 0.37 mmole/L) (Table S4 B). Homozygous carriers of the minor alleles of rs247616 (n = 516) were at 1.55 +/− 0.43 mmole/L. The groups with other allele combinations had much fewer subjects because of the negative LD between the two SNPs, and therefore could not be evaluated.

Reported rs9930761 and rs5883 allele frequencies differ between ethnic groups, while maintaining high LD and r^2^, ranging from 0% in Asians, and 7.5% in Caucasians to 12.5% in Yorubans (Table S6 for rs9930761). In a Yoruban population, rs9930761 allele frequency was reported to be 4% in subjects with low HDL levels, and 16% in those with high HDL, suggesting a large effect on HDL in this population (Table S6). Taken all results together, we conclude that rs5883 and rs9930761 have strong effects on HDL-C, independent of the upstream promoter/enhancer SNPs for which *Taq1B* typically has served as a surrogate if not suboptimal marker.

### *CETP* genotype effect on progression to event in the INVEST-GENE study

While the genotyping array contains 95 *CETP* SNPs, the present study on patients with pre-existing coronary artery disease and high blood pressure focuses on a single hypothesis, namely, whether the newly discovered splicing SNPs have clinical relevance. Even though present at relatively low allele frequency, rs5883*T*/rs9930761*C* were significantly associated with risk for an event (either MI, stroke, or death), in males (rs5883 in Caucasians, OR 2.36; 95% CI 1.29–4.3, p = 0.0051) (Table 2 and Table S5 A; other groups were too small to assess ethnic differences). As no significant association was observed in females, we propose that this effect is sex-dependent, as previously suggested for the influence of CETP variants on outcomes [13]–[15]. In contrast to the interactions between the promoter/enhancer SNPs observed with HDL-C, there was no discernible interaction with respect to outcomes, consistent with the notion that the promoter enhancer variants have no effect, or the effect is too small to be observed in this cohort. Given the relatively low allele frequency of the splicing variants and the high odds ratios, the influence of the splicing SNPs on outcomes appears to be substantial.

Elevated HDL-C levels associated with rs5883*T*/rs9930761*C* would normally be considered protective. However, this subgroup of male patients may suffer MI's with primary causes other than aberrant lipid metabolisms. Also, CETP may have distinct biological effects not reflected in overall HDL and LDL levels, including anti-inflammatory properties [32] that could have been compromised by exon 9 deletion.

In conclusion, the clinical outcome studies suggest that rs5883/rs9930761 are predictive of increased primary events (MI, stroke and death) in male at-risk patients. The results reported here support *CETP* variants as a potential disease markers and predictor of statin therapy outcome, and in evaluating CETP inhibitor drugs, such as torcetrapib [3], in the treatment of coronary artery disease.

### Limitations of the study

While the liver results strongly implicate rs5883/rs9930761 as a causative factor in CETP mRNA splicing, further molecular studies are needed to resolve the mechanism and regulation of splicing, and to identify the regulatory variant(s) affecting transcription. Also, our results leave open whether rs5883/rs9930761 are risk factors independent of statin use or affect MI risk under statin therapy in males, or both. Lastly, the important conclusion that male CAD patients with specific *CETP* genotypes may be at elevated risk of MI incidence or other outcomes has been drawn from a patient registry not specifically designed for this study, requiring independent replication.

## Materials and Methods

### Study samples

#### Human liver tissues

Frozen human liver samples (125 normal liver biopsy and autopsy samples) were obtained from The Cooperative Human Tissue Network, Midwestern and Western Divisions, which is funded by the National Cancer Institute. Other investigators may have received specimens from the same subjects. CHTN specimens are derived from material that is removed as part of routine medical care or autopsy specimens collected in accordance with operative state and local law. Every CHTN institution has obtained human subjects assurance from the Office of Human Research Protections, DHHS. The Assurance document provides agreement that the institution will comply with federal human subjects regulations. Each Division of the CHTN is approved by its local IRB to collect and distribute biospecimens. Collection to processing intervals were <24 hours.

### Whitehall II study

Between 1985 and 1988, all civil servants aged between 35 and 55 years in 20 departments in London were invited to a medical examination at their workplace [33]. Follow-up visits took place every two years. In the present analysis, *CETP* association with HDL was limited to white subjects (n = 4745) [25]. The WHII study was approved by the UCL Research Ethics Committee, and participants gave written informed consent to each aspect of the study. Ethics approval was obtained at all hospitals or institutions where participants were recruited.

### INVEST-GENES

The INternational VErapamil SR Trandolapil Study (INVEST) [34] evaluated adverse cardiovascular outcomes following randomized treatment with either an atenolol- or a verapamil-based treatment strategy in 22,576 patients aged 50 years or older, with documented CAD and essential hypertension as defined by JNC VI [34]. Primary outcomes were first occurrence of all-cause mortality, nonfatal myocardial infarction (MI), or nonfatal stroke. From 5,979 INVEST patients from 213 sites in the USA and Puerto Rico providing DNA samples, a nested case–control study was designed with 292 INVEST-GENES patients experiencing primary outcome events during follow-up (cases) and 1168 individuals who did not, frequency-matched to cases for age (by decades), sex, and race/ethnicity in a ratio of approximately 4∶1 (controls/cases), an approach shown to yield equivalent results to analyses of the entire cohort [35]. All patients provided written informed consent for participation in the main INVEST and in the genetic substudy and both studies were approved by the University of Florida Institutional Review Board.

### RNA and DNA preparation from liver tissues

RNA was extracted from 125 biopsy or autopsy liver tissues. Frozen tissue samples were pulverized under liquid nitrogen. RNA was extracted using TRIZOL ™, followed by DNase treatment and Qiagen RNeasy column purification. cDNA was generated from 1 µg purified mRNA using the Superscript II kit (Invitrogen, Carlsbad, CA) with oligo-dT and *CETP* gene-specific primers. Liver DNA was prepared by digestion of pulverized frozen liver tissue in Tris EDTA buffer containing proteinase K and SDS, followed by NaCl salting-out of proteins and ethanol precipitation [36].

### Quantitative RT-PCR (qRT-PCR) analysis of *CETP* mRNA

Real-time PCR was performed on an ABI 7000 instrument using ABI SYBR Green master mix (primer sequences in Table S1). Beta-actin and *CETP*-specific primers amplified with >99% efficiency.

### Allelic CETP mRNA expression in human liver tissues

As an accurate measure of cis-acting regulatory factors, allelic mRNA ratios were measured after conversion to cDNAs and PCR amplification, using a primer extension method (SNaPshot, Life Technologies, Foster City, CA) [30]. Allelic mRNA ratios were normalized to gDNA ratios (standardized to 1, SD ±0.03). Standard curves with cloned cDNAs representing the two alleles gave straight lines with R^2^ = 0.99 (Figure S3.). Standard deviations for each individual allelic mRNA ratio ranged from 3–8%. We also employed allele-selective qRT-PCR, which yielded similar allelic mRNA ratios compared to SNaPshot R = 0.89, (Figure S4), supporting accuracy of the results.

### Quantitative analysis of CETP Δ9 splice variant using RT-PCR with fluorescently labeled primers and splice variant-specific qRT-PCR

Splice variants were simultaneously PCR amplified using one set of splice-specific (Δ9 and long splice variant) forward primers and a common 6-FAM-fluorescently labeled reverse primer in a Sigma Ready Mix solution (Sigma Aldrich, St. Louis, MO) primers: Table S1). Liver cDNA was PCR-amplified for 25 cycles yielding amplicons of 409 bp (Δ9) and 450 bp (long), and the fluorescent peaks were analyzed on an ABI 3730 sequencer, using Gene Mapper version 3.1 (Life Technologies, Foster City, CA). Independent analysis using quantitative RT-PCR with SYBR-Green gave similar results (R^2^ = 0.86; Figure S4.). Cloned fragments of full length *CETP* and the Δ9 splice variant were used to establish linearity of the assay R^2^>0.99 (Figure S3.).

### Genotyping

Multiple methods, including TaqMan, fluorescent restriction mapping and GC clamped allele-specific PCR [37],were used to genotype 13 CETP SNPs in liver (see Table S2). These are standard methods used for low throughput genotyping. All clinical study cohorts were genotyped using the Illumina (Illumina Inc. San Diego, CA, USA) IBC Candidate Gene array, version 2 (WHII) [25] or version 3 (INVEST-GENES), representing between 49,094 (v2) to 53,831 (v3) SNPs covering ∼2,100 cardiovascular candidate loci, with 95 *CETP* SNPs [25]. The content for the custom array was chosen based on published scientific literature, cardiovascular disease pathway analysis, and recent whole-genome analysis data sets. The researchers used Illumina's iSelect Custom Genotyping BeadChip to assess the genetic diversity within pathways of approximately 2,100 genes believed to underpin primary and secondary vascular disease processes. See Table S4 for additional details. SNPs with a less than 95% call rate were excluded. Individuals with a call rate less than 95%, related samples, and population outliers were excluded using PLINK [38] and EIGENSTRAT. Hardy– Weinberg Equilibrium was evaluated using chi-squared test.

### Sequencing *CETP* exon 8 to exon 10 splice region

We sequenced a 3.1 kilobase fragment of the *CETP* exon 8–10 region in 6 livers with high or low Δ9 splice formation. Three segments of approximately 1200 bases each were PCR amplified and Sanger sequenced in both directions on an ABI 3730. The CETP sequences obtained corresponded to published DNA sequence. All variants were identified by previously assigned rs numbers.

### Statistical Methods

Statistical analysis of associations between CETP polymorphisms and allelic mRNA ratios or percent splice Δ9 splice variant was performed using the Helix Tree genetic analysis software package (Golden Helix, Inc., Bozeman, MT) [29]. Splicing was analyzed using a both Genotype and Basic Allele Tests. Allelic mRNA ratios were analyzed with genotype tests. F-Test p values are reported. Pair-wise linkage disequilibrium (LD) was determined for each combination of liver SNPs, also using Helix Tree software See Figure S5 for L.D. plot. Haplotypes were predicted with the Helix Tree estimation-maximization algorithm.

### Association between *CETP* SNPs and HDL-C in the Whitehall II study

Two (rs173539 and rs3816117) out of 13 SNPs investigated *in vitro* were not present on the Illumina IBC Candidate Gene array, version 2. These two, and additional *CETP* SNPs, were imputed from the HapMap3 and 1000 Genomes Project CEU datasets using the IMPUTEv2 software (http://mathgen.stats.ox.ac.uk/impute/impute_v2.html). *CETP* SNP association analysis with log-transformed HDL was carried out using PLINK (http://pngu.mgh.harvard.edu/purcell/plink/) [38], assuming an additive model. The additive model was used in order to maximize the prediction quality of the dependent variable from various distributions. For the additive effects of SNPs, the direction of the regression coefficient represents the effect of each extra minor allele. Analysis was performed in men and women separately with no adjustment for any covariates. A further analysis was carried out conditional on the enhancer region SNP rs247616, which itself was strongly associated with HDL levels. The Whitehall II analyses were not adjusted for use of lipid-lowering drugs because the prevalence was generally low; of 5059 total individuals from WH-II, 39 (0.8%) were taking lipid-lowering medication at the time of lipid measurement [25].

### INVEST-GENES

Baseline characteristics were compared using chi-squared test or analysis of variance. To minimize population stratification in the diverse population of INVEST, all analyses were conducted separately by race/ethnicity. For the INVEST-GENES case-control samples, adjusted odds ratios (ORs) and 95% confidence intervals (CIs) for occurrence of the primary outcome were calculated using logistic regression. Assuming alpha level of 0.05, at minor allele frequency of 6%, we have >90% power to detect a SNP main effect with OR of 2 or greater. However, in order to have >80% power to detect SNP*treatment interaction OR of 2 or greater, 4 times as many patients would be needed.

## Supporting Information

Figure S1 **CETP Gene structure, including locations of the main CETP polymorphisms determined in this study.**
(TIFF)

Figure S2 **Mfold RNA folding predictions of CETP exon 9.** The rs5883 T variant influences internal base pairing of the exon. This causes changes in nucleotide access at both 5′ and 3′ ends of the exon.
(TIFF)

Figure S3 **Allelic expression and splice assay standard curves.** Plasmid DNA containing either the A or G allele of I405V, or the normal (long splice) or Δ9 (short splice) isoform of CETP was diluted over 3 orders of magnitude. Allele specific or splice specific primers were used to amplify and quantitate the samples via Real-Time PCR in SYBR Green Master Mix (Applied Biosystems). Each point represents the average of 3 standard curves.
(TIFF)

Figure S4 **Correlation between Real-Time and fluorescent assay results.** Correlation between allele specific Real Time PCR (RT) assays and SNaPshot Primer Extension (PE) assays (panel A), or splice specific Real-Time PCR (RT) assays and fluorescent splice specific primer assays (panel B) performed on the ABI 3730. Nine samples were analyzed in duplicate.
(TIFF)

Figure S5 **Linkage Structure of Genotyped CETP SNPs in Liver.** Thirteen SNP's were genotyped in 94 livers. Pairwise LD correlation R^2^ is shown on the horizontal axis. Pairwise D′ is shown on the vertical axis. Calculated and graphed using Helix Tree Genetic Analysis Software Package.
(TIF)

Table S1 **PCR primers and amplicons for rs993076 and the CETP splice variant (other primers are available on request).** rs5883 was determined using preselected commercial TaqMan and PCR probes (Life Technologies, Foster City, CA).
(DOCX)

Table S2 **Table of polymorphisms in the** ***CETP*** **locus genotyped in the liver study, and genotyping methods.** Not all of the SNPs were analyzed in each liver, so that the allele frequencies reflect some selection bias and may not represent allele frequencies in the clinical groups in this study.
(DOCX)

Table S3 **Estimated** ***CETP*** **haplotypes constructed from 5 SNPs genotyped in 44 liver samples (calculated with HelixTree).** rs5883 and rs9930761 are in complete LD in the liver samples tested for all SNPs in these tissues. The EM probability represents ambiguity in calling the individual haplotypes.
(DOCX)

Table S4 **A**. Association p values between *CETP* SNPs and log-transformed HDL-C levels in the Whitehall II study (4,744 subjects, males plus females). SNPs with available rs id number and unadjusted p value <0.001 were included. **B**. Mean HDL-C levels grouped by genotype for rs247616 and rs5883 in all subjects. **C**. Significant interaction between effects of rs5883 and rs247616 on HDL-C levels.
(DOCX)

Table S5 **A**. Allele associations between *CETP* polymorphisms and primary outcomes in the INVEST-GENES study (464 males and 402 females, all Caucasians) using an additive model. The SNPs in this table are sorted by chromosomal location. The p values are unadjusted. **B**. Minor allele frequencies and LD between rs9930761 and rs5883 in the INVEST cohort.
(DOCX)

Table S6 **Published allele frequencies of rs9930761 in various populations (** http://www.ncbi.nlm.nih.gov/projects/SNP/snp_ref.cgi?rs=9930761 **) (September 2010).**
(DOCX)
