# Supplementary figures and images for: Cholesteryl Ester Transfer Protein (CETP) Polymorphisms Affect mRNA Splicing, HDL Levels, and Sex-Dependent Cardiovascular Risk
Source: PLoS One. 2012 Mar 5;7(3):e31930. doi: 10.1371/journal.pone.0031930 (PMC3293889; doi:10.1371/journal.pone.0031930)

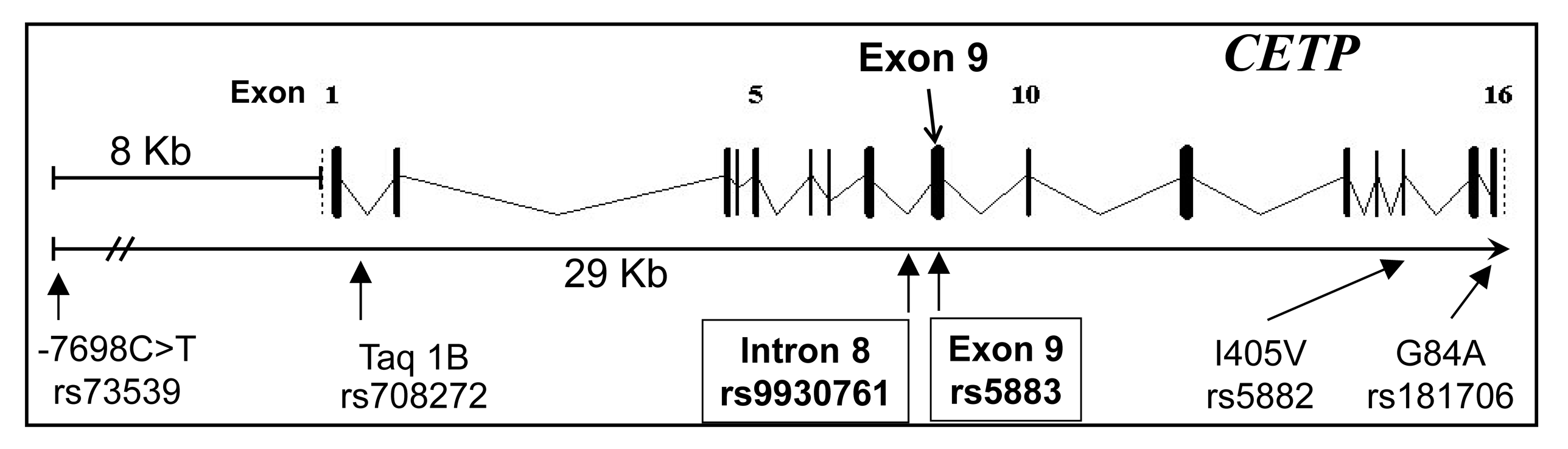

Supplement: Figure S1 — CETP Gene structure, including locations of the main CETP polymorphisms determined in this study. (TIFF) [file pone.0031930.s001.tiff]

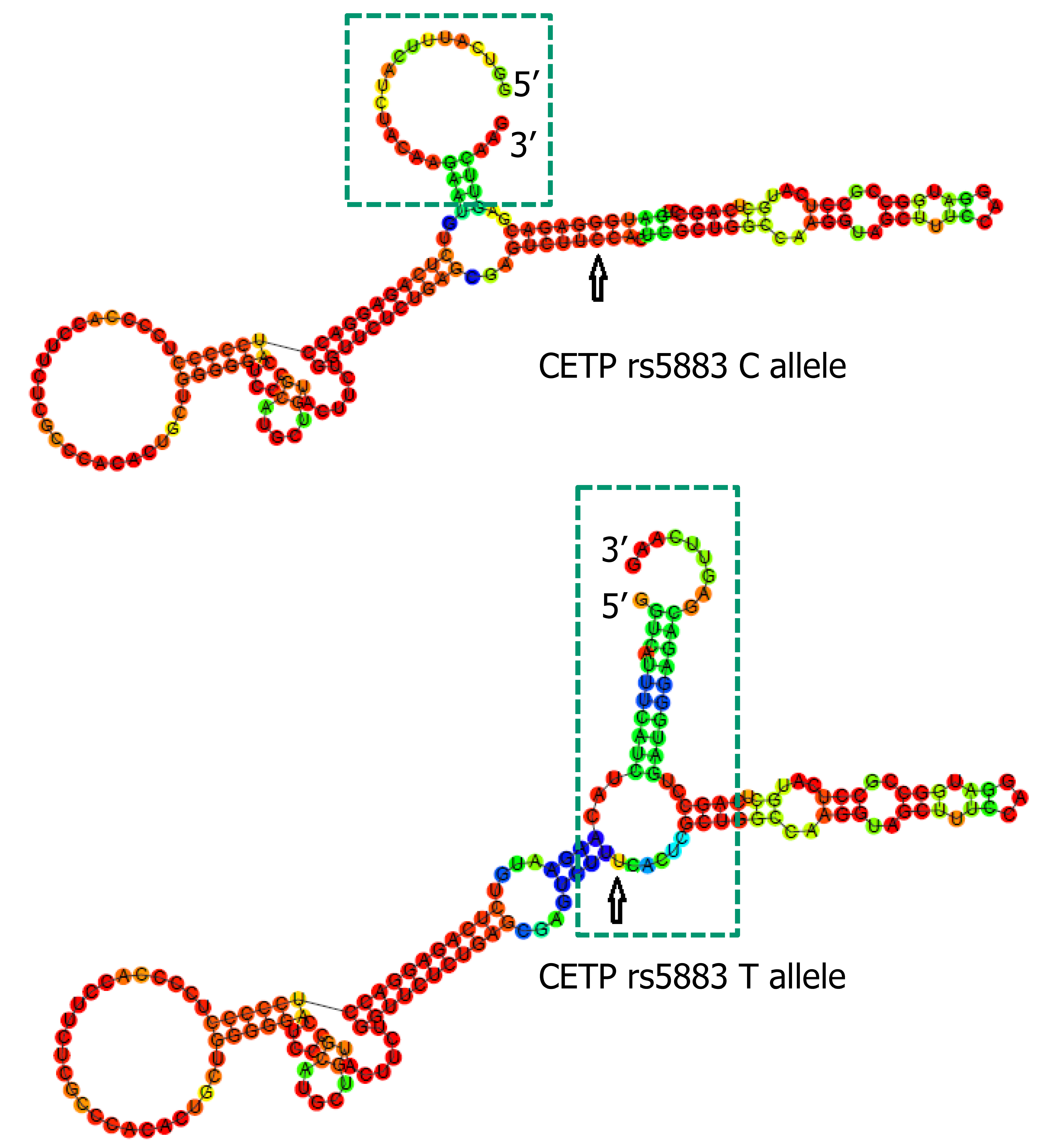

Supplement: Figure S2 — Mfold RNA folding predictions of CETP exon 9. The rs5883 T variant influences internal base pairing of the exon. This causes changes in nucleotide access at both 5′ and 3′ ends of the exon. (TIFF) [file pone.0031930.s002.tiff]

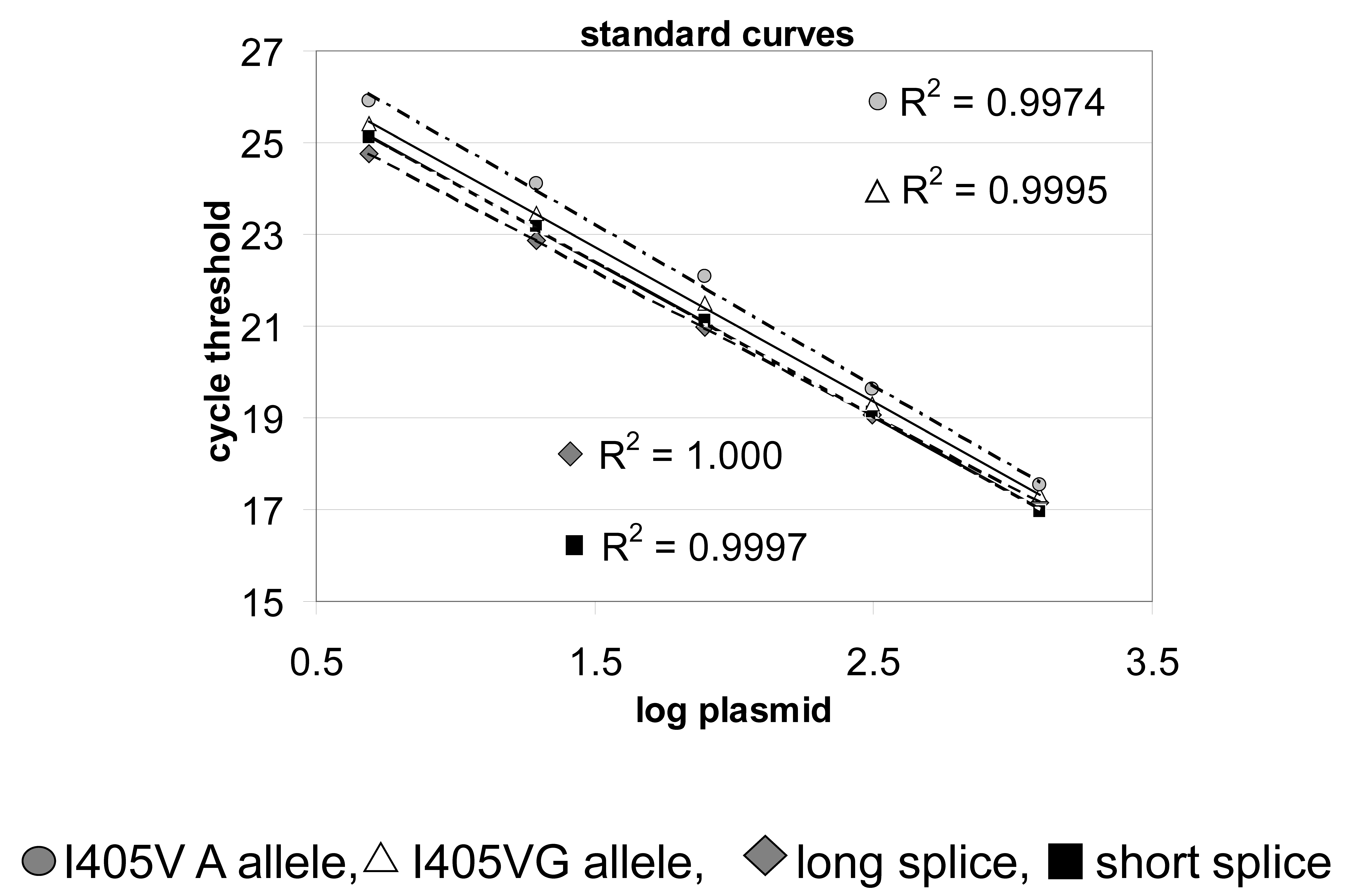

Supplement: Figure S3 — Allelic expression and splice assay standard curves. Plasmid DNA containing either the A or G allele of I405V, or the normal (long splice) or Δ9 (short splice) isoform of CETP was diluted over 3 orders of magnitude. Allele specific or splice specific primers were used to amplify and quantitate the samples via Real-Time PCR in SYBR Green Master Mix (Applied Biosystems). Each point represents the average of 3 standard curves. (TIFF) [file pone.0031930.s003.tiff]

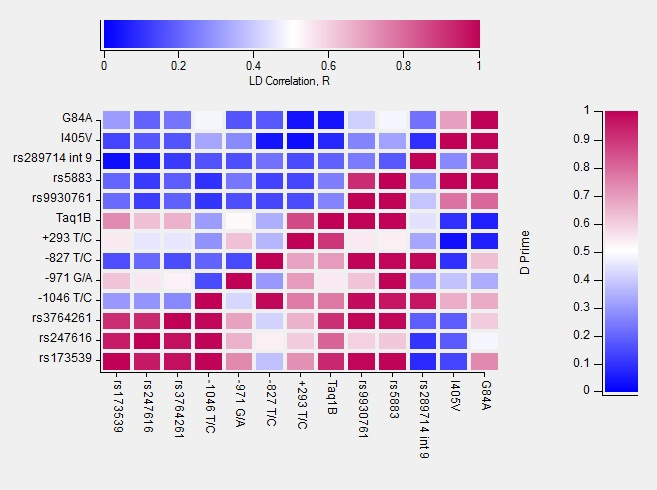

Supplement: Figure S5 — Linkage Structure of Genotyped CETP SNPs in Liver. Thirteen SNP's were genotyped in 94 livers. Pairwise LD correlation R2 is shown on the horizontal axis. Pairwise D′ is shown on the vertical axis. Calculated and graphed using Helix Tree Genetic Analysis Software Package. (TIF) [file pone.0031930.s005.tif]
