# Supplementary material for: Cholesteryl Ester Transfer Protein (CETP) Polymorphisms Affect mRNA Splicing, HDL Levels, and Sex-Dependent Cardiovascular Risk
Source: PLoS One. 2012 Mar 5;7(3):e31930. doi: 10.1371/journal.pone.0031930 (PMC3293889; doi:10.1371/journal.pone.0031930)
Supplement: Table S1 — PCR primers and amplicons for rs993076 and the CETP splice variant (other primers are available on request). rs5883 was determined using preselected commercial TaqMan and PCR probes (Life Technologies, Foster City, CA). (DOCX) [file pone.0031930.s006.docx]

**Table S1.** PCR primers and amplicons for rs993076 and the CETP splice variant (other primers are available on request). rs5883 was determined using preselected commercial TaqMan and PCR probes (Life Technologies, Foster City, CA).

| **Primer Name** | **Sequence** | **Purpose** |
| --- | --- | --- |
| **ex8-10spF3*** | GGAGTCCCATCACAAGGCAG | Short Δ9 splice specific |
| **ex9_10spF** | CCTGATGGGAGACGAGTTCAA | Long splice specific |
| **genex14R*** | TACGAGACATGACCTCAGGGA | Common splice reverse (FAM label) |
| **CETP rs9930761-192F-FAM** | TTGAATGAGTGAAAGCCCCG | Fluorescent genotyping |
| **CETP rs9930761-342R** | GACGCTGGCTGGACCCT | Reverse genotyping primer |
| **CETP rs5883 E9F** | CACCTTCTCGCCCACACTG | Taqman PCR probes |
| **CETP rs5883 E9R** | GAACTCGTCTCCCATCAGGC | Taqman PCR probes |
| **CETP ex8-10 seq-54F** | CCCGTCATCACAGCCTCCTA | Sequencing amplicon |
| **CETP ex8-10 seq-1254R** | CATTCAGCCCAGAGTGCAGAT | Sequencing amplicon |
| **CETP ex8-10 seq-1065F** | GCCCTGACTAATGTCGTTACTTGA | Sequencing amplicon |
| **CETP ex8-10 seq-2268R** | CCCATGTTGCCCAGGCT | Sequencing amplicon |
| **CETP ex8-10 seq-1928F** | TGCATGCACCGGTAATCAGAA | Sequencing amplicon |
| **CETP ex8-10 seq-3128R** | CCCAGGTCTCCAGCACTGC | Sequencing amplicon |
| **CETP ex8-10 seq-2320F** | CGGCGGTGCATACCTATAGTC | Sequencing amplicon |

***F:** primer in forward direction; **R**: primer in reverse direction
