# Supplementary material for: Cholesteryl Ester Transfer Protein (CETP) Polymorphisms Affect mRNA Splicing, HDL Levels, and Sex-Dependent Cardiovascular Risk
Source: PLoS One. 2012 Mar 5;7(3):e31930. doi: 10.1371/journal.pone.0031930 (PMC3293889; doi:10.1371/journal.pone.0031930)
Supplement: Table S2 — Table of polymorphisms in the CETP locus genotyped in the liver study, and genotyping methods. Not all of the SNPs were analyzed in each liver, so that the allele frequencies reflect some selection bias and may not represent allele frequencies in the clinical groups in this study. (DOCX) [file pone.0031930.s007.docx]

**Table S2. Polymorphisms in the *CETP* locus genotyped in the liver study, and genotyping methods.** Not all of the SNPs were analyzed in each liver, so that the allele frequencies reflect some selection bias and may not represent allele frequencies in the clinical groups in this study.

| **rs** | **Location***** | **Chr pos** | **Base** | **Minor allele frequency CAU** | **Amino acid** |
| --- | --- | --- | --- | --- | --- |
| **rs173539** | -7698 | 55545545 | *C>T* | 35.5% | n.a. |
| **rs247616** | -6152 | 55547091 | *C>T* | 33.0% | n.a. |
| **rs3764261** | -2418 | 55550825 | *G>T* | 33.6% | n.a. |
| **rs12708968** | -1046 | 55552197 | *T>C* | 12.5% | n.a. |
| **rs4783961** | -971 | 55552272 | *G>A* | 45.7% | n.a. |
| **rs4783962** | -827 | 55552415 | *C>T* | 19.2% | n.a. |
| **rs3816117** | Intr1 +293 | 55553659 | *C>T* | 43.5% | n.a. |
| **rs708272** | Intr1 +429 (*Taq1B*) | 55553789 | *T>C* | 47.8% | n.a. |
| **rs9930761** | **Intr8 -40** | **55564693** | *T>C* | 7% | n.a. |
| **rs5883** | **Exon9 +121** | **55564854** | *C>T* | 6% | synonymous |
| **rs289714** | Intr9 +29 | 55564952 | *T>C* | 14.7% | n.a. |
| **rs5882** | Exon14 +1264 (*I405V*) | 55573593 | *A>G* | 48.0% | Ile --> Val |
| **rs1801706** | 3’UTR +84 (*G84A*) | 55575163 | *G>A* | 20.0% | n.a. |

An Applied Biosystems ABI 7000 instrument was used for genotyping with SNaPshot and TaqMan MGB ™ probes (*Taq1B*, rs5583, *I405V*, rs1800774 (Intron12), and *G84A*). rs9935061 was genotyped with a *BsoBI*  restriction enzyme assay. Primers were designed using Primer Express version 2.0 (all genotyping reagents: Life Technologies, Foster City, CA). rs173539 was genotyped with a HaeIII restriction enzyme assay. The HEX labeled forward primer sequence used is CCTGTGGTCCCAGTTACTTAGGA. The reverse primer is CCCCAATCTGTAGTCTTTGCCA. rs247616 was genotyped using a Taq1 restriction enzyme assay. The FAM labeled forward primer is GACTCAACAACAGGGCCACA. The reverse primer is ACTTCGATTAAAAGAGTTCTGGAGATGGGTT. rs3764261 was genotyped using the GC clamp method. Forward allele specific primers used were CGTCCCGCGCCGCCCCTGTCGGTAGGCATCTTGG (Tm 91.7°C) specific for the G allele and ACCTGTCGGTAGGCATCAGGT (Tm 66.8°C) specific for the T allele. A common reverse primer (CAGGGCAATCAAGGCATCC) was used.
