# Supplementary material for: Cholesteryl Ester Transfer Protein (CETP) Polymorphisms Affect mRNA Splicing, HDL Levels, and Sex-Dependent Cardiovascular Risk
Source: PLoS One. 2012 Mar 5;7(3):e31930. doi: 10.1371/journal.pone.0031930 (PMC3293889; doi:10.1371/journal.pone.0031930)
Supplement: Table S3 — Estimated CETP haplotypes constructed from 5 SNPs genotyped in 44 liver samples (calculated with HelixTree). rs5883 and rs9930761 are in complete LD in the liver samples tested for all SNPs in these tissues. The EM probability represents ambiguity in calling the individual haplotypes. (DOCX) [file pone.0031930.s008.docx]

**Table S3. Estimated *CETP* haplotypes constructed from 5 SNPs genotyped in 44 liver samples (calculated with HelixTree).** rs5883 and rs9930761 are in complete LD in the liver samples tested for all SNPs in these tissues. The EM probability represents ambiguity in calling the individual haplotypes. Most tissues carrying the minor *T* allele of rs5883 yielded unambiguous or high confidence haplotypes, showing that the minor *T* allele of rs5883 is predominantly associated with the major alleles of rs173539 and *Taq1B*, but with the minor alleles of *I405V* and *G84A* (haplotype *C_G_****C****_G_A*), The single exception being assigned *C_A_****T****_G_A* has low EM probability so that the phasing cannot be determined with confidence.

| **SNP order** | **Base** | **Haplotype** | **Frequency in livers (%)** |
| --- | --- | --- | --- |
| **rs173539** | C/T | C_G_C_A_G | 28.4 |
| **Taq1B** | G/A | C_A_C_G_A | 5.7 |
| **rs9930761*** | C/T | C_A_C_A_G | 3.4 |
| **I405V** | A/G | C_G_C_G_G | 1.1 |
| **G84A** | G/A | C_G_**T**_G_A | 9.1 |
|  |  | C_A_**T**_G_A** | 1.1 |
|  |  | T_A_C_A_G | 21.6 |
|  |  | T_A_C_G_A | 9.0 |
|  |  | T_A_C_G_G | 11.4 |

*rs9930761*T>C* is interchangeable with rs5883 in this group of tissues

**Low EM probability (0.36)
