# Supplementary material for: Cholesteryl Ester Transfer Protein (CETP) Polymorphisms Affect mRNA Splicing, HDL Levels, and Sex-Dependent Cardiovascular Risk
Source: PLoS One. 2012 Mar 5;7(3):e31930. doi: 10.1371/journal.pone.0031930 (PMC3293889; doi:10.1371/journal.pone.0031930)
Supplement: Table S4 — A. Association p values between CETP SNPs and log-transformed HDL-C levels in the Whitehall II study (4,744 subjects, males plus females). SNPs with available rs id number and unadjusted p value <0.001 were included. B. Mean HDL-C levels grouped by genotype for rs247616 and rs5883 in all subjects. C. Significant interaction between effects of rs5883 and rs247616 on HDL-C levels. (DOCX) [file pone.0031930.s009.docx]

**Table S4 A. Association p values between *CETP* SNPs and log-transformed HDL-C levels in the Whitehall II study** (4,744 subjects, males plus females). SNPs with available rs id number and unadjusted p value <0.001 were included. Promoter/enhancer SNP rs247616 and splice SNP rs9930761 are highlighted in red. The SNP shown in blue is a representative SNPs in high LD with other promoter/enhancer SNPs, and with strong association to log-transformed HDL, also highlighted in the Dutch case-control statin study and in INVES-GENES (Suppl. Tables 6 and 7). SNPs are presented in the order of their P values. SNPs were genotyped using Illumina (Illumina Inc. San Diego, CA, USA) IBC Candidate Gene array, version 2 (WHII) or version 3 ( Utrecht Cardiovascular Pharmacogenetics (UCP) cohort and INVEST-GENES)** (see also Suppl. Tales 6 and 7).

| **SNP** | **BP** | **Sample Size** | **Beta** | **Std. Error** | **R^2^** | **Unadjusted P-value** |
| --- | --- | --- | --- | --- | --- | --- |
| **rs17231506** | 55552029 | 4730 | 0.06838 | 0.006065 | 0.02618 | 4.11E-29 |
| **rs173539** | 55545545 | 4680 | 0.06814 | 0.00605 | 0.02641 | 4.65E-29 |
| **rs247616** | 55547091 | 4513 | 0.06909 | 0.006154 | 0.02719 | 7.18E-29 |
| **rs3764261** | 55550825 | 4722 | 0.06785 | 0.006045 | 0.026 | 7.21E-29 |
| **rs12149545** | 55550662 | 4682 | 0.06752 | 0.006089 | 0.0256 | 3.19E-28 |
| **rs247617** | 55548217 | 4641 | 0.06635 | 0.006062 | 0.02517 | 1.52E-27 |
| **rs183130** | 55548864 | 4592 | 0.06824 | 0.006296 | 0.02495 | 4.80E-27 |
| **rs708272** | 55553789 | 4708 | 0.06175 | 0.005728 | 0.0241 | 8.77E-27 |
| **rs711752** | 55553712 | 4724 | 0.06138 | 0.005743 | 0.02362 | 2.31E-26 |
| **rs1532625** | 55562802 | 4701 | 0.05938 | 0.005712 | 0.02248 | 4.83E-25 |
| **rs7205804** | 55562390 | 4697 | 0.05929 | 0.005707 | 0.02247 | 5.16E-25 |
| **rs1532624** | 55562980 | 4721 | 0.05927 | 0.005716 | 0.02228 | 6.35E-25 |
| **rs1800775** | 55552737 | 4723 | 0.0588 | 0.005694 | 0.02209 | 9.83E-25 |
| **rs3816117** | 55553659 | 4612 | 0.05874 | 0.005739 | 0.02222 | 2.53E-24 |
| **rs11508026** | 55556829 | 4717 | 0.05862 | 0.005754 | 0.02154 | 3.99E-24 |
| **rs56228609** | 55545266 | 4185 | 0.06431 | 0.006311 | 0.02422 | 4.23E-24 |
| **rs34620476** | 55554150 | 4434 | 0.05934 | 0.005874 | 0.0225 | 9.89E-24 |
| **rs12444012** | 55558939 | 4516 | 0.05834 | 0.00581 | 0.02185 | 1.76E-23 |
| **rs1864163** | 55554734 | 4738 | -0.06485 | 0.006479 | 0.02071 | 2.37E-23 |
| **rs4784741** | 55558717 | 4512 | 0.0582 | 0.005813 | 0.02174 | 2.39E-23 |
| **rs7499892** | 55564091 | 4731 | -0.07005 | 0.007455 | 0.01833 | 8.56E-21 |
| **rs8045855** | 55558197 | 4263 | -0.08004 | 0.00859 | 0.01997 | 1.84E-20 |
| **rs11076175** | 55563879 | 4726 | -0.06871 | 0.007465 | 0.01762 | 5.07E-20 |
| **rs9939224** | 55560233 | 4739 | -0.06395 | 0.007036 | 0.01714 | 1.45E-19 |
| **rs12708967** | 55550712 | 4728 | -0.06486 | 0.007156 | 0.01709 | 1.80E-19 |
| **rs289713** | 55564330 | 4436 | -0.07087 | 0.007888 | 0.01788 | 3.77E-19 |
| **rs12720926** | 55556419 | 3920 | 0.05649 | 0.006314 | 0.02002 | 5.57E-19 |
| **rs12720922** | 55558386 | 4742 | -0.06506 | 0.007413 | 0.01599 | 2.33E-18 |
| **rs12923459** | 55547331 | 4479 | -0.04993 | 0.005819 | 0.01618 | 1.29E-17 |
| **rs7203984** | 55556759 | 4728 | -0.06175 | 0.007259 | 0.01508 | 2.35E-17 |
| **rs9929488** | 55556073 | 4216 | -0.05325 | 0.007069 | 0.01328 | 6.07E-14 |
| **rs28888131** | 55549125 | 4230 | -0.06368 | 0.008456 | 0.01324 | 6.14E-14 |
| **rs12720918** | 55551713 | 4718 | -0.04397 | 0.006288 | 0.01026 | 3.09E-12 |
| **rs9926440** | 55560164 | 3223 | -0.05125 | 0.007467 | 0.01442 | 7.98E-12 |
| **rs12920974** | 55550526 | 4595 | -0.04215 | 0.006412 | 0.00932 | 5.47E-11 |
| **rs289718** | 55567433 | 4685 | 0.03868 | 0.006158 | 0.008357 | 3.64E-10 |
| **rs11076176** | 55564947 | 4737 | -0.04878 | 0.007806 | 0.00818 | 4.49E-10 |
| **rs289716** | 55566877 | 4728 | 0.03658 | 0.006186 | 0.007346 | 3.58E-09 |
| **rs12720889** | 55570064 | 4731 | 0.03652 | 0.006223 | 0.007231 | 4.69E-09 |
| **rs289719** | 55567442 | 4692 | 0.03621 | 0.006212 | 0.007193 | 5.94E-09 |
| **rs5880** | 55572592 | 4743 | -0.07403 | 0.01278 | 0.007022 | 7.49E-09 |
| **rs289714** | 55564952 | 4743 | -0.04379 | 0.007626 | 0.006907 | 9.93E-09 |
| **rs289717** | 55566889 | 4733 | -0.03363 | 0.00603 | 0.006535 | 2.56E-08 |
| **rs4784744** | 55568686 | 4736 | -0.03325 | 0.00602 | 0.006403 | 3.50E-08 |
| **rs289743** | 55575297 | 4699 | 0.03439 | 0.006263 | 0.006377 | 4.22E-08 |
| **rs291044** | 55568953 | 4735 | -0.0328 | 0.006022 | 0.006229 | 5.39E-08 |
| **rs1800777** | 55574820 | 4743 | -0.08198 | 0.01509 | 0.00619 | 5.78E-08 |
| **rs289744** | 55575603 | 4689 | 0.03335 | 0.006288 | 0.005967 | 1.18E-07 |
| **rs4784745** | 55572376 | 4738 | -0.03159 | 0.006037 | 0.005748 | 1.74E-07 |
| **rs289741** | 55574975 | 4694 | 0.03273 | 0.006279 | 0.005756 | 1.95E-07 |
| **rs291043** | 55570200 | 4549 | -0.03138 | 0.006125 | 0.005741 | 3.12E-07 |
| **rs5882** | 55573593 | 4718 | 0.02988 | 0.006193 | 0.004911 | 1.45E-06 |
| **rs708273** | 55557450 | 4730 | -0.02934 | 0.006336 | 0.004514 | 3.75E-06 |
| **rs12597002** | 55559905 | 4727 | -0.02894 | 0.006336 | 0.004397 | 5.05E-06 |
| **rs5883*** | 55564854 | 4742 | 0.05616 | 0.0124 | 0.004308 | 6.09E-06 |
| **rs60545348** | 55559486 | 4697 | -0.02832 | 0.006317 | 0.004263 | 7.52E-06 |
| **rs4369653** | 55555052 | 4527 | -0.02705 | 0.006428 | 0.003898 | 2.62E-05 |
| **rs9923854** | 55574503 | 4738 | 0.03946 | 0.009587 | 0.003564 | 3.92E-05 |
| **rs1801706*** | 55575163 | 4741 | 0.03049 | 0.007492 | 0.003483 | 4.78E-05 |
| **rs1800776** | 55552735 | 4743 | -0.04047 | 0.01069 | 0.003014 | 0.000155 |
| **rs12720917** | 55576893 | 4736 | 0.02854 | 0.007829 | 0.002799 | 0.00027 |
| **rs820299** | 55557785 | 4728 | -0.02045 | 0.005953 | 0.002491 | 0.000597 |
| **rs12720873*** | 55563573 | 4744 | 0.05494 | 0.01665 | 0.002291 | 0.000976 |
| **rs9930761** | 55564693 | 4740 | 0.03681 | 0.01138 | 0.002204 | 0.001224 |
| **rs11644475*** | 55565153 | 4575 | 0.06006 | 0.01864 | 0.002266 | 0.00128 |

**_________________________________________________________________________**

*LD with rs9930761 in Caucasians (r^2^): 0.881 (rs5883), 0.649 (rs12720873), 0.563 (rs11644475), and 0.126 (rs1801706, *G84A*) (LD from 1,000 genome database)

** Talmud PJ, Drenos F, Shah S, Shah T, Palmen J, Verzilli C, Gaunt TR, Pallas J, Lovering R, Li K, Casas JP, Sofat R, Kumari M, Rodriguez S, Johnson T, Newhouse SJ, Dominiczak A, Samani NJ, Caulfield M, Sever P, Stanton A, Shields DC, Padmanabhan S, Melander O, Hastie C, Delles C, Ebrahim S, Marmot MG, Smith GD, Lawlor DA, Munroe PB, Day IN, Kivimaki M, Whittaker J, Humphries SE, Hingorani AD. Gene-centric association signals for lipids and apolipoproteins identified via the humancvd beadchip. *Am J Hum Genet*. 2009;85:628-642.

**Processes for SNP selection:**

Group 1 (n = 435 loci); genes and regions with a high likelihood of functional significance, including established mediators of vascular disease, loci derived from GWAS and those shown to be associated with phenotypes of interest. Tag SNPs for these loci were selected to capture known variation with MAF>0.02 and an r2 of at least 0.8 in HapMap populations and SeattleSNPs where available (for formal description, see Calculation of Coverage section below).

Group 2 (n = 1,349 loci); candidate loci that are potentially involved in phenotypes of interest or established loci that required very large numbers of tagging SNPs. SNPs for these loci were selected for MAFs>0.05 with an r2 of at least 0.5 in HapMap populations and SeattleSNPs where available.

Group 3 (n = 232 loci); comprised mainly of the larger genes (>100 kb) which were of lower interest a priori to the consortium investigators. Only non-synonymous SNPs (nsSNPs) and known functional variants of MAF>0.01 were captured for these loci.

Assays for specific SNPs of known or putative functionality and those shown to be highly associated with vascular disease from literature searching were directly ‘forced’ into the array content, with the aim of facilitating more powerful downstream meta-analyses with previously published data. nsSNPs and known functional variants of MAF>0.01 were selected where possible for all genes of interest.

SNPs from Group 1 and 2 loci were first chosen using the TAGGER software. Assays for SNPs in Group 1 loci were designed to be inclusive of the intronic, exonic, untranslated regions (UTRs) and 5 kb of the proximal promoter regions derived from NCBI build 35 with intronic, exonic and flanking UTRs covered for the ‘Group 2’ loci. This approach generated a set of tag SNPs and multimarker predictors that capture variation in the four HapMap populations (CEU, Centre d'Etude du Polymorphisme Humain collection; CHB, Han Chinese in Beijing, China; JPT, Japanese individuals from Tokyo, YRI, Yoruba from Ibadan, Nigeria; HapMap Data release 21/phase II July 2006 on NCBI build 35, dbSNP build 125). Where available, we also employed SeattleSNPs (http://pga.gs.washington.edu) and Environmental Genome Project (EGP), (http://egp.gs.washington.edu) resequencing data to identify additional tags, not represented in the HapMap populations, using ldSelect. We choose SNPs that were observed at least twice in unrelated individuals.

SNP selection information taken from: *Keating BJ, Tischfield S, Murray SS, Bhangale T, Price TS, et al. (2008) Concept, Design and Implementation of a Cardiovascular Gene-Centric 50 K SNP Array for Large-Scale Genomic Association Studies. PLoS ONE 3(10): e3583. doi:10.1371/journal.pone.0003583*

**Table S4 B.** Mean HDL-C levels grouped by genotype for rs247616 and rs5883 in all subjects. Because of the relatively low allele frequency of rs5883, some of the subgroups do not contain a sufficient number of subjects to obtain reliable mean HDL-C levels (*e.g*., the *CC/TT* haplotype (n=3). However, carriers heterozygous for both SNPs (*TC/*TC; n=142) show a substantial increase in HDL-C. In the vast majority of these subjects, the minor *T* allele of the two SNPs will be on opposite haplotypes.

| **rs247616** | **rs5883** | **rs247616**  **# minor**  **alleles** | **rs5883**  **# minor**  **alleles** | **Total**  **# minor**  **alleles** | **# subjects** | **Mean HLD-C**  **(mmol/L)** | **SD** |
| --- | --- | --- | --- | --- | --- | --- | --- |
| **CC** | **CC** | 0 | 0 | 0 | 1651 | 1.35 | 0.37 |
| **CC** | **TC** | 0 | 1 | 1 | 310 | 1.43 | 0.39 |
| **TC** | **CC** | 1 | 0 | 1 | 1865 | 1.45 | 0.41 |
| **CC** | **TT** | 0 | 2 | 2 | 20 | 1.47 | 0.37 |
| **TT** | **CC** | 2 | 0 | 2 | 516 | 1.55 | 0.43 |
| **TC** | **TC** | 1 | 1 | 2 | 142 | 1.67 | 0.48 |
| **TC** | **TT** | 1 | 2 | 3 | 3 | 2.02 | 0.39 |
| **TT** | **TC** | 2 | 1 | 3 | 3 | 2.30 | 0.32 |
| **TT** | **TT** | 2 | 2 | 4 | 0 | NA | NA |

**Table S4 C.** Significant interaction between effects of rs5883 and rs247616 on HDL-C levels.

(lgxhdl ~ b0 + b1.rs5883 + b2.rs247616 + b3.rs5883*rs247616 + e)

| **SNP** | **Beta** | **SE** | **P value** |
| --- | --- | --- | --- |
| **rs5883** | 0.056 | 0.012 | 6.09E-06 |
| **rs247616** | 0.069 | 0.0062 | 7.18E-29 |
| **rs5883*rs247616** | 0.093 | 0.026 | 0.00033 |
