# Supplementary material for: Cholesteryl Ester Transfer Protein (CETP) Polymorphisms Affect mRNA Splicing, HDL Levels, and Sex-Dependent Cardiovascular Risk
Source: PLoS One. 2012 Mar 5;7(3):e31930. doi: 10.1371/journal.pone.0031930 (PMC3293889; doi:10.1371/journal.pone.0031930)
Supplement: Table S5 — A. Allele associations between CETP polymorphisms and primary outcomes in the INVEST-GENES study (464 males and 402 females, all Caucasians) using an additive model. The SNPs in this table are sorted by chromosomal location. The p values are unadjusted. B. Minor allele frequencies and LD between rs9930761 and rs5883 in the INVEST cohort. (DOCX) [file pone.0031930.s010.docx]

**Table S5 A.** **Allele associations between *CETP* polymorphisms and primary outcomes in the INVEST-GENES study (464 males and 402 females, all Caucasians) using an additive model.** The SNPs in this table are sorted by chromosomal location. The p values are unadjusted. SNPs are presented in the order they appear in the genomic sequence.

| **SNP** | **position** | **p_male** | **p_female** |
| --- | --- | --- | --- |
| rs6499861 | 55548996 | 0.69 | 0.020 |
| rs6499863 | 55549518 | 0.97 | 0.032 |
| rs12708967 | 55550712 | 0.012 | 0.77 |
| rs3764261 | 55550825 | 0.592 | 0.067 |
| rs13332526 | 55551636 |  | 0.47 |
| rs12447924 | 55551693 | 0.045 | 0.72 |
| rs12720918 | 55551713 | 0.18 | 0.98 |
| rs13332571 | 55551739 |  | 0.47 |
| rs17231506 | 55552029 | 0.58 | 0.048 |
| rs12708968 | 55552320 | 0.16 | 0.91 |
| rs4783961 | 55552395 | 0.021 | 0.15 |
| rs4783962 | 55552539 | 0.055 | 0.73 |
| rs17237883 | 55552625 | 0.047 | 0.30 |
| rs1800776 | 55552735 | 0.84 | 0.66 |
| rs1800775 | 55552737 | 0.37 | 0.054 |
| rs17231520 | 55553328 | 0.98 | 0.83 |
| rs5884 | 55553458 | 0.30 | 0.80 |
| rs17231534 | 55553605 | 0.018 | 0.55 |
| rs711752 | 55553712 | 0.89 | 0.13 |
| rs708272 | 55553789 | 0.86 | 0.12 |
| rs9935228 | 55554573 |  | 0.61 |
| rs12720906 | 55556262 | 0.98 | 0.47 |
| rs7203984 | 55556759 | 0.78 | 0.017 |
| rs11508026 | 55556829 | 0.96 | 0.18 |
| rs708273 | 55557450 | 0.028 | 0.71 |
| rs820299 | 55557785 | 0.61 | 0.39 |
| rs12720922 | 55558386 | 0.52 | 0.0075 |
| rs12597002 | 55559905 | 0.065 | 0.77 |
| rs9939224 | 55560233 | 0.54 | 0.062 |
| rs11076174 | 55560647 | 0.50 | 0.20 |
| rs891141 | 55561224 | 0.24 | 0.97 |
| rs891142 | 55561478 | 0.28 | 0.98 |
| rs12720861 | 55561960 | 0.61 | 0.30 |
| rs1532625 | 55562802 | 0.97 | 0.41 |
| rs12720925 | 55562926 | 0.61 |  |
| rs1532624 | 55562980 | 0.97 | 0.14 |
| rs12708974 | 55563051 | 0.26 | 0.64 |
| rs12597250 | 55563121 | 0.57 | 0.58 |
| rs12720872 | 55563383 | 0.47 | 0.307 |
| rs12720873* | 55563573 | 0.17 | 0.65 |
| rs11076175 | 55563879 | 0.83 | 0.0098 |

*LD with rs9930761 (r^2^): 0.649 (rs12720873)

and 0.126 (rs1801706, *G84A*) (1,000 genome database)

| **SNP** | **position** | **p_male** | **p_female** |
| --- | --- | --- | --- |
| rs7499892 | 55564091 | 0.86 | 0.020 |
| **rs9930761** | **55564693** | **0.0019** | **0.90** |
| **rs5883** | **55564854** | **0.0018** | **0.73** |
| rs11076176 | 55564947 | 0.10 | 0.056 |
| rs289714 | 55564952 | 0.966 | 0.093 |
| rs158477 | 55565111 | 0.94 | 0.30 |
| rs289715 | 55566009 | 0.12 | 0.19 |
| rs12720877 | 55566701 | 0.61 | 0.10 |
| rs289716 | 55566877 | 0.81 | 0.33 |
| rs289717 | 55566889 | 0.62 | 0.44 |
| rs12708976 | 55567012 |  | 0.47 |
| rs12720942 | 55567109 |  | 0.47 |
| rs736274 | 55567270 | 0.16 | 0.28 |
| rs12720939 | 55567300 | 0.61 | 0.31 |
| rs289718 | 55567433 | 0.86 | 0.30 |
| rs289719 | 55567442 | 0.87 | 0.26 |
| rs2033254 | 55567486 | 0.49 | 0.78 |
| rs4784744 | 55568686 | 0.62 | 0.46 |
| rs12720898 | 55568744 | 0.27 | 0.87 |
| rs291044 | 55568953 | 0.62 | 0.44 |
| rs891144 | 55569437 | 0.57 | 0.50 |
| rs12708979 | 55569855 | 0.084 | 0.0024 |
| rs12708980 | 55569880 | 0.57 | 0.81 |
| rs12720889 | 55570064 | 0.77 | 0.52 |
| rs12708984 | 55571923 | 0.42 | 0.029 |
| rs4784745 | 55572376 | 0.58 | 0.30 |
| rs7192120 | 55572585 |  | 0.049 |
| rs5880 | 55572592 | 0.44 | 0.22 |
| rs7195984 | 55572964 | 0.47 | 0.58 |
| rs7196174 | 55573045 | 0.28 | 0.34 |
| rs1800774 | 55573046 | 0.23 | 0.88 |
| rs5882 | 55573593 | 0.23 | 0.22 |
| rs8045701 | 55573803 | 0.98 | 0.30 |
| rs12720882 | 55573815 | 0.43 | 0.10 |
| rs289740 | 55574451 | 0.47 | 0.47 |
| rs9923854 | 55574503 | 0.041 | 0.71 |
| rs1800777 | 55574820 | 0.44 | 0.90 |
| rs1801706* | 55575163 | 0.29 | 0.99 |
| rs289742 | 55575263 | 0.064 | 0.15 |
| rs289743 | 55575297 | 0.63 | 0.25 |
| rs12720917 | 55576893 | 0.85 | 0.27 |

**Table S5 B**.  **Minor allele frequencies and LD between rs9930761 and rs5883 in the INVEST cohort.**

|  | **rs9930761** | **rs5883** |  |  |
| --- | --- | --- | --- | --- |
|  | **MAF** | **MAF** | **D'** | **r2** |
| **White** | 0.073 | 0.060 | 1 | 0.81 |
| **Black** | 0.105 | 0.099 | 0.97 | 0.89 |
| **hisp** | 0.053 | 0.046 | 0.94 | 0.77 |
