# Supplementary material for: Cholesteryl Ester Transfer Protein (CETP) Polymorphisms Affect mRNA Splicing, HDL Levels, and Sex-Dependent Cardiovascular Risk
Source: PLoS One. 2012 Mar 5;7(3):e31930. doi: 10.1371/journal.pone.0031930 (PMC3293889; doi:10.1371/journal.pone.0031930)
Supplement: Table S6 — Published allele frequencies of rs9930761 in various populations ( http://www.ncbi.nlm.nih.gov/projects/SNP/snp_ref.cgi?rs=9930761 ) (September 2010). (DOCX) [file pone.0031930.s011.docx]

**Table S6.**

**Published allele frequencies of rs9930761 in various populations**

(<http://www.ncbi.nlm.nih.gov/projects/SNP/snp_ref.cgi?rs=9930761>) (September 2010).

| ***Populations*** | ***Minor allele (C) frequency*** | ***Number of samples*** |
| --- | --- | --- |
| **HapMap CEU*** | **0.075** | **120** |
| **HapMap JPT/HCB*** | **0.000** | **90** |
| **HapMap YRI*** | **0.125** | **120** |
| **PF** CEU elderly***** | **0.100** | **44** |
| **PF** CEU Low HDL****** | **0.050** | **52** |
| **PF** AA Low HDL****** | **0.040** | **42** |
| **PF** AA High HDL****** | **0.160** | **50** |

CEU: Central European; JPT/HCB: Japanese/Han Chinese; YRI: Yoruban; CEU: Caucasians; AA: African Americans

**PF: Pfizer study (Submitter: Albert B. Seymour, PhD, Pfizer Global R&D, Pharmacogenomics, 1 Eastern Point Road, MS_8118D-3006, Groton, CT 06340)

***>90 years old; ****low: =/<30 mg/dl CEU and =/<37 mg/dl AA; high: >74 mg/dl
